# TRPM8 Channel Promotes the Osteogenic Differentiation in Human Bone Marrow Mesenchymal Stem Cells

**DOI:** 10.3389/fcell.2021.592946

**Published:** 2021-02-04

**Authors:** Juan C. Henao, Adriana Grismaldo, Alfonso Barreto, Viviana M. Rodríguez-Pardo, Claudia Camila Mejía-Cruz, Efrain Leal-Garcia, Rafael Pérez-Núñez, Patricio Rojas, Ramón Latorre, Ingrid Carvacho, Yolima P. Torres

**Affiliations:** ^1^Departamento de Nutrición y Bioquímica, Facultad de Ciencias, Pontificia Universidad Javeriana, Bogotá, Colombia; ^2^Grupo de Inmunobiología y Biología Celular, Departamento de Microbiología, Facultad de Ciencias, Pontificia Universidad Javeriana, Bogotá, Colombia; ^3^Departamento de Ortopedia y Traumatología, Facultad de Medicina, Pontificia Universidad Javeriana, Bogotá, Colombia; ^4^Médico Ortopedista y Traumatólogo, Consulta Privada, Bogotá, Colombia; ^5^Departamento de Biología, Facultad de Química y Biología, Universidad de Santiago de Chile, Santiago, Chile; ^6^Centro Interdisciplinario de Neurociencia de Valparaíso, Facultad de Ciencias, Universidad de Valparaíso, Valparaíso, Chile; ^7^Department of Biology and Chemistry, Faculty of Basic Sciences, Universidad Católica del Maule, Talca, Chile

**Keywords:** TRP channels, bone marrow mesenchymal stem cells, osteogenic differentiation, mesenchymal stem cell, TRPM8 channel

## Abstract

Various families of ion channels have been characterized in mesenchymal stem cells (MSCs), including some members of transient receptor potential (TRP) channels family. TRP channels are involved in critical cellular processes as differentiation and cell proliferation. Here, we analyzed the expression of TRPM8 channel in human bone marrow MSCs (hBM-MSCs), and its relation with osteogenic differentiation. Patch-clamp recordings showed that hBM-MSCs expressed outwardly rectifying currents which were increased by exposure to 500 μM menthol and were partially inhibited by 10 μM of BCTC, a TRPM8 channels antagonist. Additionally, we have found the expression of TRPM8 by RT-PCR and western blot. We also explored the TRPM8 localization in hBM-MSCs by immunofluorescence using confocal microscopy. Remarkably, hBM-MSCs treatment with 100 μM of menthol or 10 μM of icilin, TRPM8 agonists, increases osteogenic differentiation. Conversely, 20 μM of BCTC, induced a decrease of osteogenic differentiation. These results suggest that TRPM8 channels are functionally active in hBM-MSCs and have a role in cell differentiation.

## Introduction

Mesenchymal stem cells (MSCs) have a high proliferative capacity and the ability to differentiate *in vitro* into multiple cell types, including neurons, osteocytes, chondrocytes, and adipocytes (Bobis et al., 2006; Müller et al., 2008; Sundelacruz et al., 2008; Collins et al., 2010; Serakinci et al., 2014). MSCs also support processes related to hematopoiesis and regulation of the immune system *in vivo* (Rodríguez-Pardo and Vernot, 2013; Rodríguez-Pardo et al., 2013; Mejía-Cruz et al., 2019). Cell differentiation is a complex process involving diverse factors, including hormones, cytokines, and ion channels expression (Ramírez-Ponce et al., 2002; Sundelacruz et al., 2008). Understanding the role of ion channels in MSCs will thus help to clarify the mechanisms responsible for regulating the differentiation process, contributing to new strategies in regenerative medicine. Expression of cation channels in MSCs and their roles in the proliferation and differentiation have been studied previously (Heubach et al., 2004; Yang and Huang, 2005; Zahanich et al., 2005; Li et al., 2006; Bai et al., 2007; Ding et al., 2012; Wen et al., 2012; You et al., 2013; Zhang et al., 2014). Voltage-gated potassium channels have been related to adipogenic differentiation of hMSCs (You et al., 2013) and their inhibition, decreased MSC proliferation (Heubach et al., 2004; Deng et al., 2007). Additionally, treatment of hBM-MSCs with tetrodotoxin increased cell proliferation, suggesting a role for sodium channels in this process (Ding et al., 2012), while blocking calcium (Ca^2+^) channels inhibited cell proliferation and osteogenic differentiation (Ding et al., 2012; Wen et al., 2012).

The Transient Receptor Potential (TRP) channels are a family of mostly non-selective ubiquitously expressed cationic channels. TRP channels family is composed of six sub-families: canonical (TRPCs), vanilloid (TRPVs), melastatin (TRPMs), mucolipin (TRPMLs), polycystic subfamily (TRPP) and ankyrin (TRPA) (Wu et al., 2010).

Members of TRP channels family are expressed in excitable tissues, where they have been related to physiological processes such as nociception, temperature, pressure and vision signaling (Gees et al., 2010; Latorre et al., 2011). In immune cells, TRP channels play critical roles in the regulation of various cell functions including chemotaxis in neutrophils (TRPC6) (McMeekin et al., 2006) and cytokine production in T-cells (TRPM2) (Knowles et al., 2011). Some TRP members are over expressed in cancer cells. For example, TRPV1 is increased in prostate, colon and pancreas cancer cells, and TRPM8 has been found in breast, colon, lung and prostate cancer cells (Clapham, 2003; Prevarskaya et al., 2007; Wondergem et al., 2008; Wondergem and Bartley, 2009; Shapovalov et al., 2011; Bauer et al., 2012). Expression of TRP channels have been reported in mouse BM-MSCs and hDPSC (Cheng et al., 2010; Cui et al., 2014). In bone marrow-derived MSCs and human dental pulp stem cells (hDPSCs), TRPM7 channels are involved in proliferation, migration, and differentiation, having a critical function in osteogenic differentiation and dental-pulp repair (Cheng et al., 2010; Cui et al., 2014). TRPC1, 2, 4 and 6 channels are expressed in rabbit MSCs, and they have been reported to contribute to cell proliferation (Everaerts et al., 2010; Nilius and Owsianik, 2010; Torossian et al., 2010; Nilius and Voets, 2013). Here, we examined the expression of TRPM8 channels in hBM-MSCs. TRPM8 is a member of the TRP channels subfamily melastatin, and it is activated by temperatures below 28°C and by the plant derivative agonist menthol and the synthetic agent icilin. Other less potent natural TRPM8 agonists are eucalyptol, linalool and geraniol (Voets et al., 2007; González-Muñiz et al., 2019). Additionally, TRPM8 is blocked by non-selective agents as 4-(3-Chloro-2-pyridinyl)-N-[4-(1,1-dimethylethyl) phenyl]-1-piperazinecarboxamide (BCTC) and 2-amitoethoxydiphenhyl borate (2-APB) (Voets et al., 2007). TRPM8, as non-selective channel, is permeable to sodium, potassium, cesium and calcium, and its activation induces currents with outward rectification (Hui et al., 2005; Latorre et al., 2011). The channel was cloned by two independent groups with different experimental strategies, who identified TRPM8 (also called CMR1) as a putative cold sensor in primary sensory afferents (McKemy et al., 2002; Peier et al., 2002). TRPM8 has been reported to have a role regulating cell proliferation and differentiation in diverse cells such as oral squamous carcinoma and prostatic cells (Bidaux et al., 2007; Okamoto et al., 2012). In 2007, three independent groups generated *trpm8* null mouse lines (Bautista et al., 2007; Colburn et al., 2007; Dhaka et al., 2007). TRPM8-KO mice had severe impairment in cold sensation including deficits and lacked allodynia and cold analgesia (Dhaka et al., 2007).

Despite the increased information about TRPM8 function in different cell types, the role of the channels in human mesenchymal stem cells during osteogenic differentiation has not been explored. A better knowledge about the function of TRPM8 channels during the differentiation process may further improve our understanding of the potentials hBM-MSCs used in regenerative medicine.

## Materials and Methods

### Reagents

Menthol (Sigma), Icilin (sigma) and 4-(3-Chloro-2-pyridinyl)-N-[4-(1,1-dimethylethyl) phenyl]-1-piperazinecarboxamide (BCTC) (Sigma) were solubilized in dimethyl sulfoxide (DMSO) to prepare a stock solution. Work solutions were prepared in the bath solution (for electrophysiological recordings) or culture medium (differentiation assays). In all experiments, final concentration of DMSO was <0.1%. StemPro Adipogenesis Differentiation Kit and StemPro Osteogenesis Differentiation Kit were purchased to Gibco. LipidTox, Goat anti-rabbit IgG, Anti Rabbit Alexa Fluor 488 and Lipofectamine were purchase to Invitrogen. The anti-TRPM8 antibody was from Alomone labs (ACC-049). RNeasy Micro Kit, SuperScript III, Bicinchoninic acid kit and protease/phosphatase inhibitor cocktail were purchase from Qiagen, Life Technologies, Sigma and Cell signaling, respectively. 100 bp DNA ladder, Polyvinylidene difluoride membrane (PVDF), SuperSignal West Dura Extended Duration Substrate and RIPA buffer were from Thermo Scientific.

### hBM-MSC Isolation and Culture Conditions of Human Bone Marrow MSC

To obtain MSC, bone marrow (BM) samples were obtained with the support of the Department of Orthopedics and Traumatology of Hospital Universitario San Ignacio (Bogota, Colombia) from five volunteer donors (undergoing hip replacement surgery) after signing the informed consent form approved by the Hospital Ethics Committee according to optimal criteria for bone marrow donors to isolate hBM-MSC [Acta No 18 (2014/154)] (Barreto-Durán et al., 2018). BM-MSC were isolated and cultured as previously published (Rodríguez-Pardo and Vernot, 2013; Rodríguez-Pardo et al., 2013; Pardo-perez et al., 2018; Mejía-Cruz et al., 2019), and they were used in passages 3–5. BM-MSC phenotype was assessed by flow cytometry using a Guava cytometer (Guava easyCyte; Millipore). hBM-MSC were characterized according to the expression of the surface markers CD73 (BD 561260), CD105 (BD 561443), without the expression of CD34 (Beckman Coulter IM2472) and CD45 (Beckman Coulter IM1833). 1 × 10^5^ cells were centrifuged and suspended in 300μl of PBS (phosphate-buffered saline)-FBS (Fetal bovine serum) 2% with the antibody. After 30 min, cells were washed and suspended in 300 μl of PBS and marker expression was analyzed by flow cytometry. Guava Incyte Software was used for data analysis.

### Osteogenic and Adipogenic Differentiation Capacity of hBM-MSC *in vitro*

hBM-MSC were analyzed for the differentiation capacity. 3 × 10^5^ cells were plated in 12 well plates and after 16 h of adhesion the differentiation medium was added: StemPro® Osteogenesis Differentiation Kit for osteogenic differentiation and StemPro® Adipogenesis Differentiation for adipogenic differentiation. The medium was changed every third day. After 14 days the differentiation, medium was removed and cell differentiation was analyzed. For adipogenic differentiation, cells were dyed with LipidTOX (H34476), following manufacturing recommendations and analyzed by flow cytometry. For osteogenic differentiation, cells were fixed with formaldehyde 4% during 15 min and stained with alizarine red 2% (AMRESCO 9436). Cells were observed under a microscope and pictures of the wells were taken with a 20X objective (Zeiss Axiocam ERc 5s Mobile Stand-Alone Color Microscope Camera). The differentiated area was analyzed using ImageJ. Cells without differentiation media were used as negative control.

### Modulation of Osteogenic Differentiation by TRPM8 Channels Modulators

To analyze the effect of TRPM8 channel modulators, osteogenic differentiation was carried out in the presence of Menthol 100 μM, Icilin 10 μM or BCTC 20 μM. Medium (StemPro® Osteogenesis Differentiation Kit) containing the drugs was changed every third day during the differentiation process. Cells incubated with regular media were used as a negative control. Osteogenic cell differentiation was determined by alizarin red staining (as previously described). The differentiated area was calculated with the ImageJ software, and changes in expression of ALPL (Alkaline phosphatase) were measured by qPCR by the comparative CT method. Statistical analyses were performed using GraphPad Prism (One-way ANOVA).

### Analysis of TRPM8 Channel Expression

Total mRNA was isolated from hBM-MSCs using a RNeasy Micro Kit (Qiagen 74004) according to the manufacturer's instructions. The mRNA was purified and reverse transcribed using SuperScript IIIMR (Gibco-BRL, Life technologies). Conventional PCR was performed using the kitGoTaq polymerase (Promega M3001) using the primers: forward: 5′ CAGCGCTGGAGGTGGATATTC 3′; reverse: 5′ CACACACAGTGGCTTGGACTC (Size 144 bp; Accession number XM_024453134) 3′. The fragment was amplified by PCR using an amplification kit (Roche). Ribosomal protein 27 (*RPL27*) was used as a housekeeping gene (forward: 5′: ATCGCCAAGAGATCAAAGATAA 3′; reverse 5′ TCTGAAGACATCCTTATTGACG 3′; 120bp). Total mRNA from HEK293 cells transfected with TRPM8 (using lipofectamine 2000; Thermo Fisher 11668027) was used as a positive control and total mRNA from HEK293FT cells was used as a negative control of expression. The resulting fragment was visualized by 2% agarose gel electrophoresis. To check the size of the transcript, 100-bp DNA ladder was used.

Western blot, flow cytometry and confocal microscopy assays were carried out to analyze protein expression. For Western blot, hBM-MSC were seeded in 12 well plates and grown to 80% confluency. Cells were washed twice with PBS and then lysed on ice with buffer RIPA (Sigma-Aldrich 20-188) and protease inhibitors (Sigma-Aldrich p18690). The lysates were centrifuged at 14,000 g for 5 min and the supernatant was stored at −80°C. BCA Protein Assay Kit (Sigma) was used to determine protein concentration. After heat denaturation, proteins were separated in 10% SDS-PAGE gel and transferred to PVDF membrane (88518 Thermo Scientific). Membrane was blocked for 1 h with 10 mM Tris-HCl; pH 8.0, 150 mM NaCl, Twin (TBS-T) 0.1, 2% Bovine serum albumin (BSA). PVDF membrane was incubated with primary antibody 1:1,000 dilution (ACC-049, Alomone Labs) at 4°C overnight. Primary antibody was removed and samples were rinsed three times with TBS-T. Secondary antibody 1:1,000 (Goat Anti Rabbit HRP, Invitrogen ab 10183) was added and samples were incubated for 1 h in the dark at room temperature (RT). Blots were analyzed using the chemiluminescence substrate Supersignal West Dura Extended Duration Substrate (Thermo Fischer 34076) and film CL-Xposure (Thermo Fischer 34088) for 5 min. HEK cells transfected with TRPM8 channel (using lipofectamine 2000; Thermo Fisher 11668027) were used as a positive control and untransfected cells were used as a negative control.

For flow cytometry assays, cells at 80–90% confluence were detached with trypsin-EDTA, centrifuged at 360 g for 5 min, and suspended in DMEM at a density of 3 × 10^5^cells/ml. Cells were fixed with 4% formaldehyde for 15 min and centrifuged at 360 g for 5 min. The pellet was suspended in methanol and left overnight at −20°C. Cells were washed in PBS–FBS 2%, centrifuged at 360 g for 5 min, and incubated with primary antibody (ACC-049, Alomone Labs) 1: 100 overnight at 4°C. After incubation, cells were rinsed with PBS-FBS 2%, centrifuged at 360 g for 5 min and the supernatant was removed, incubated with secondary antibody Alexa fluor 488 (A-11094, Thermo Fisher) 1:400 for 1 h and washed twice with PBS-FBS 2%. Samples were acquired using a Guava easyCyte cytometer (Millipore) and analyzed with Guava Incyte Software. HEK cells transfected with TRPM8 channel (using lipofectamine 2000; Thermo Fisher 11668027) were used as a positive control and untransfected cells were used as negative control. Statistical analyses were performed using GraphPad Prism (One-way ANOVA).

In order to carry out confocal microscopy assays, 1 × 10^5^ cells were seeded in petri dish glass bottom 20 mm. After 24 h, cells were washed with PBS, fixed using 4% paraformaldehyde and incubated with PBS-Triton 0.01% for 1 h to permeabilize the cells. Cells were incubated with TBS-BSA 2% for 1 h to block non-specific binding and then with 1/100 of rabbit polyclonal antibody TRPM8 specific (ACC-049, Alomone Labs) was added. After 16 h, cells were washed with TBS-BSA 2% and incubated with the secondary antibody Alexa Fluor 488 anti-rabbit 1/400 (A-11094, Thermo Fischer) in TBS-BSA 2% at RT during 30 min. After rising with TBS, nuclei were counterstained with DAPI 300 nM for 5 min (D1306, Thermo fisher). TRPM8 expression was analyzed from imagens taken with a confocal microscopy (Olympus fluo View 1000) using laser lines 405, 488, and 633 nm. Negative controls were performed without primary antibody. Acquisition parameters were fixed using negative control as a baseline reference. Resolution images of 640 × 640 pixels were acquired using UPLSAPO 60X 1.35 NA oil immersion objective.

To study the localization of TRPM8 protein in intracellular organelles, we plated 2 × 10^4^ cells in a petri dish glass bottom. After 24 h cells were rinsed twice with PBS and incubated for 30 min with Mitotracker 500 nM (Thermo Fisher, M22425), Lisotracker 150 nM (Thermo Fisher, L12492) or 2.5 μg/mL of mouse monoclonal antibody calnexin specific (Thermo Fisher, AF18) followed by appropriated secondary antibody. Cells were fixed with 4% paraformaldehyde during 20 min, washed with PBS for 5 min and permeabilized with TBS-Triton 0.01%. Cells were washed with TBS, incubated with TBS-BSA 2% to block non-specific binding and left overnight with 1/100 of rabbit polyclonal antibody TRPM8 specific (ACC-049, Alomone Labs). Later, cells were washed twice with TBS-BSA 2% and incubated at RT with secondary antibody Alexa Fluor 488 anti-rabbit (A-11094, Thermo Fischer) 1/400 in TBS-BSA 2%. Finally, petri dishes were washed with TBS and protein expression was analyzed using a confocal microscope (Olympus fluo View 1000). Control experiments were performed without primary antibody. Colocalization of TRPM8 in different organelles was estimated using Pearson's correlation index and overlap coefficient obtained through the Image pro plus program (Dunn et al., 2011). The result was obtained from a projection of several z focal planes and then analyzed using a single focal plane representative. The selection of the analysis area was made taking into account the regions only marked with the probes (where the organelle was located), fluorescence events of TRPM8/fluorescence events of the organelle of interest were analyzed.

### Osteogenic Differentiation Analysis by Quantitative Real Time-PCR

Total RNA was extracted from hMB-MSC using Trizol-LS (Invitrogen 15596018). Differentiated cells were washed with PBS and 200 μl of Trizol-LS were added and mechanical lysis was performed for 5 min at RT. Two hundred microliters chloroform was added to each well and samples were centrifuged at 12,000 g at 4°C during 15 min. The aqueous phase was recovered and 500 μl of isopropanol 99.9% were added. Tubes were incubated 10 min at 4°C and centrifuged at 12,000 g and 4°C for 15 min. Pellet was rinsed with 500 μl ethanol 75% and centrifuged at 7,500 g and 4°C for 5 min. Pellet was dried and suspended in RNAse and DNAse free water. 500 ng/μl of RNA was used to cDNA synthesis following the manufacturer's instruction of SuperScript III Reverse Transcriptase (ThermoFisher 18080044). This cDNA was used as a template in reactions using the kit SensiFAST SYBR No-ROX (Bioline, BIO-98005), according to the manufacturer's instruction. The reaction was carried out in a program of 95°C for 2 min and then cycled 39 times at 95°C for 15 s, 62°C for 30 s and 72°C for 20s. The assays were performed in three technical replicates. The following primers were used for Alkaline phosphatase (ALPL) gen: forward 5′-CCCGCTTTAACCAGTGCAAC-3′; reverse 5′-GAGCTGCGTAGCGATGTCC-3′ (Hu et al., 2015). Glyceraldehyde-3-phosphate dehydrogenase (GAPDH) (forward 5′-CAGAGTTAAAAGCAGCCCTGGT-3, reverse 5′ GAAGGTGAAGGTCGGAGTCAAC−3′) was used as a housekeeping gene for the normalization of data. The fold changes in mRNA expression were calculated by normalization of the cycle threshold (C_t_) value of target genes. The Ct cut-off was 40. Statistical analyses were performed using GraphPad Prism (One-way ANOVA).

### Electrophysiology Assays to Analyze TRPM8 Currents

Whole-cell currents were recorded using the patch-clamp technique at 27–28°C. hBM-MSC were trypsinized and seeded onto glass coverslips 30–60 min before assays. Bath solution contained (in mM): 140 NaCl, 4.5 KCl, 2 CaCl_2_, 1 MgCl_2_, and 10 [4-(2-hydroxyethyl)-1-piperazineethanesulfonic acid] HEPES. The pipette solution contained (in mM): 140 CsCl, 2 ethylene glycol tetraacetic acid (EGTA), and 10 HEPES. pH was adjusted to 7.3–7.4. Pipettes were made from borosilicate glass capillaries (WPI, Inc; 1B150F-4), pulled in a horizontal micropipette puller (P-97, Sutter Instruments) and heat polished (MF-830, Narishige). Pipette resistance was 3–5 MΩ and seal resistance was > 1.5 GΩ. The cell mean capacitance was 27 ± 10 pF SEM (*n* = 3). Current recordings were acquired using an EPC-7 amplifier (HEKA), filtered at 1/5 of the acquisition rate and sampled with an A/D converter (NI-PCIe-6351; National Instruments). Acquisition software was developed in the LabView programming environment (National Instruments) by Dr. Patricio Orio (CINV, Valparaiso, Chile). Data analysis was performed with Clampfit 9 (Axon Instruments) software. Currents were activated by voltage ramp protocol from −100 to 200 mV (600 ms, every 2 s) with a holding potential (HP) of 0 mV. The voltage step protocol was also used with pulses between −100 and +150 mV in 10 mV increments with a duration of 45 ms. Currents were recorded before and after the addition of menthol 500 μM and BCTC 10 μM. Statistical analyses were performed using GraphPad Prism (One-way ANOVA).

### Statistical Analysis

Data are expressed as means ± SD of *n* independent measurements. The normality of data was determined by shapiro wilk test. Statistical comparison was performed by paired Student's *t*-test in electrophysiology data and unpaired Student's *t*-test in differentiation data tests. In all cases, statistical significance was assumed with *P*-values < 0.05.

## Results

### Immunophenotype and *in vitro* Differentiation Capacity of Isolated hBM-MSC

**Figure 1:**
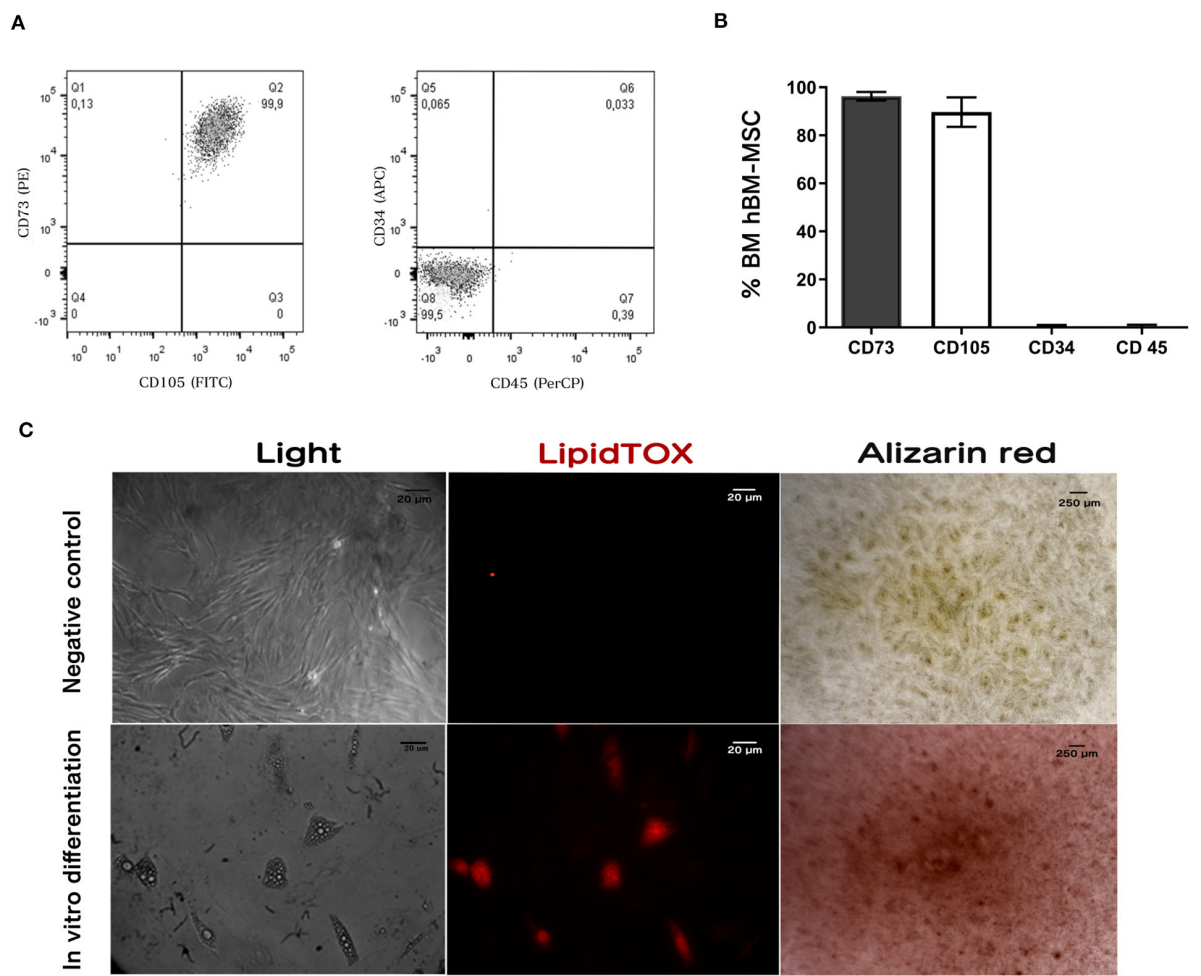
hBM-MSC characterization. Immunophenotyping by flow cytometry: Dot plots **(A)** showing antigen expression in freshly hBM-MSC. Cells do not express hematopoietic antigens CD34 and CD45 and co-express CD73 and CD105. **(B)** Antigen expression quantification using flow cytometry. **(C)** Differentiation assay: Cultured hBM-hMSC was induced to differentiate into adipocytes and osteoblast. Adipogenic differentiation was demonstrated by lipid vacuole detection with the lipid stain LipidTOX (Middle panel); and Ca^2+^ deposits in osteoblast differentiation was detected with Alizarin red (right panel). Results shown represent three independent experiments done in triplicate (*n* = 3).

Characterization showed that 96 ± 2% and 90 ± 5% of hBM-MSC) were positive for CD73 and CD105, respectively. Confirming their mesenchymal phenotype, <1% were positive for CD34 and CD45 expression as shown in Figures 1A,B. Data are mean ± SD (*n* = 3) (Dominici et al., 2006; Horwitz et al., 2007; Brinchmann, 2008).

To assay differentiation capacity to adipocyte and osteoblast, hBM-MSCs were treated with adipogenic (StemPro Adipogenesis Differentiation Kit, Invitrogen) and osteogenic (StemPro Osteogenesis Differentiation Kit, Invitrogen) differentiation media. We observed that hBM-MSCs treated with adipogenic media produced vacuoles detected with LipidTOX (Figure 1C, central panel). Furthermore, after treating hBM-MSCs with the osteogenic differentiation medium, we observed the formation of calcium deposits measured by alizarin red staining (Figure 1C, right panel). These results indicate that hBM-MSCs can differentiate to both lineages, as has been previously reported (Figure 1C) (*n* = 3).

### hBM-MSC Express Endogenous TRPM8 Channels

**Figure 2:**
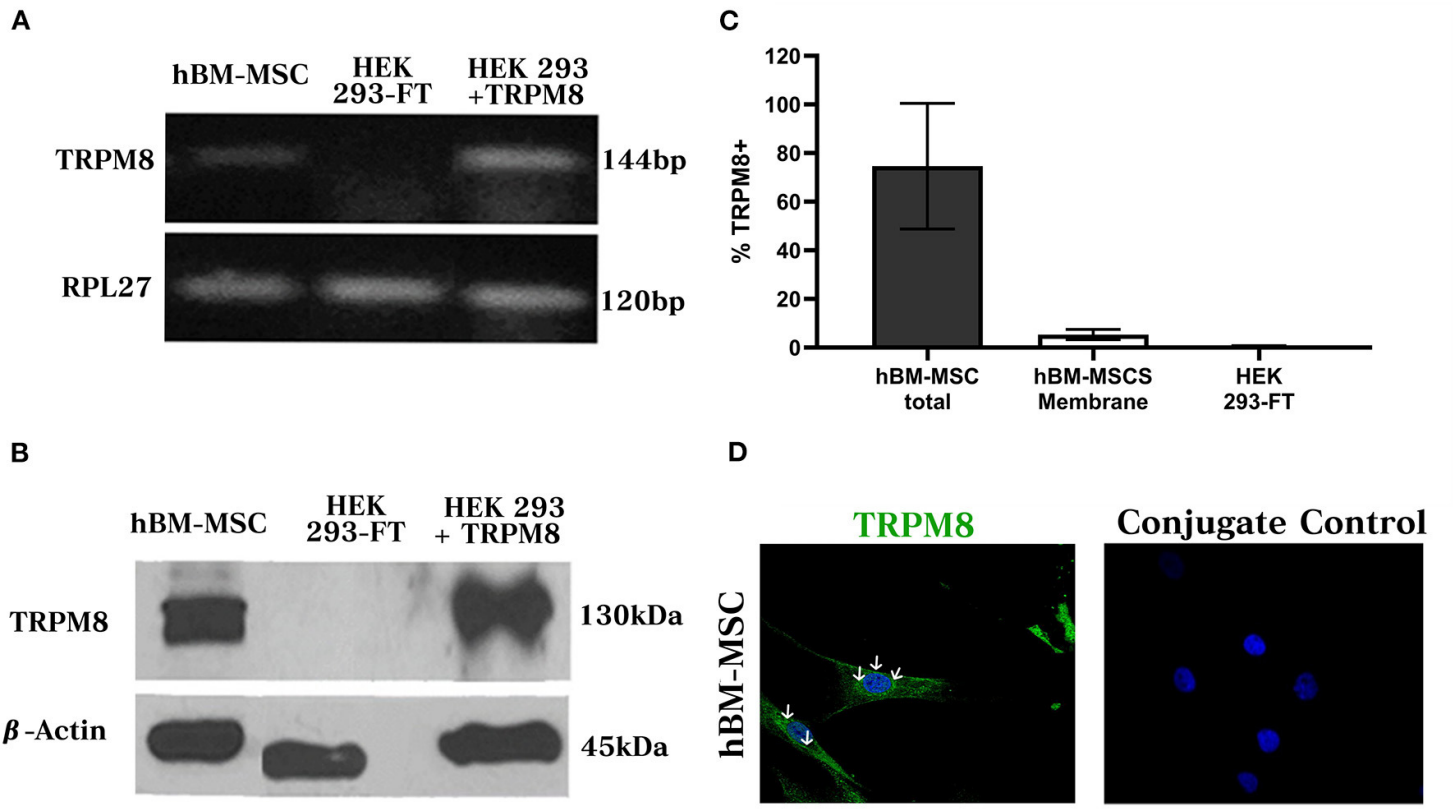
TRPM8 channel expression in hBM-MSCs. **(A)** RT-PCR from total hBM-MSCs RNA. cDNA fragment corresponding to TRPM8 transcript (144 bp) in hBM-MSCs is shown. As controls we used HEK cells transfected with TRPM8 and non-transfected cells, respectively. RPL 27 (Name of the Ribosomal proteinL27, 120 bp) is the housekeeping gene (*n* = 3). **(B)** Western blot detected TRPM8 protein in hBM-MSCs and cells transfected with TRPM8 (130 kDa). β-actin (45kDA) was used as a load control. As a negative control we used untransfected cells. **(C)** Percentage of TRPM8 total and membrane expression in hBM-MSCs and control cells transfection (*n* = 3). **(D)** TRPM8 expression pattern in permeabilized hBM-MSCs (left panel) and control of unspecific secondary antibody binding (right panel) (*n* = 4). The images presented in the figure shown confocal images of Alexa fluor 488 (TRPM8, green) and DAPI (blue).

In order to evaluate TRPM8 mRNA expression in hBM-MSC, we used conventional RT-PCR and we amplified a transcript of the expected molecular size (126 bp) (Figure 2A) (*n* = 3). The same fragment was identified in HEK293 cells transfected with TRPM8 cDNA but not in untransfected cells, indicating the specificity of this PCR product. In all cases, a signal for the amplification of the *RPL27* housekeeping gene was detected. Western blot (WB) analysis demonstrated the presence of TRPM8 in total protein extract of hBM-MSC, with a band of ~130 kDa, similar to the one observed in TRPM8 transfected cells (Figure 2B). β-actin was used as a loading control (42 kDa). No signal was found in the control untransfected cells. These findings were confirmed further by flow cytometry in permeabilized and non-permeabilized cells. We found that 78% ± 20 of hBM-MSC expressed the TRPM8 protein in both intracellular and cell membrane and 5.4% ± 2.1 expressed the protein only in the plasma membrane (PM) (Figure 2C) (*n* = 3). No signal was observed in control untransfected cells.

**Figure 3:**
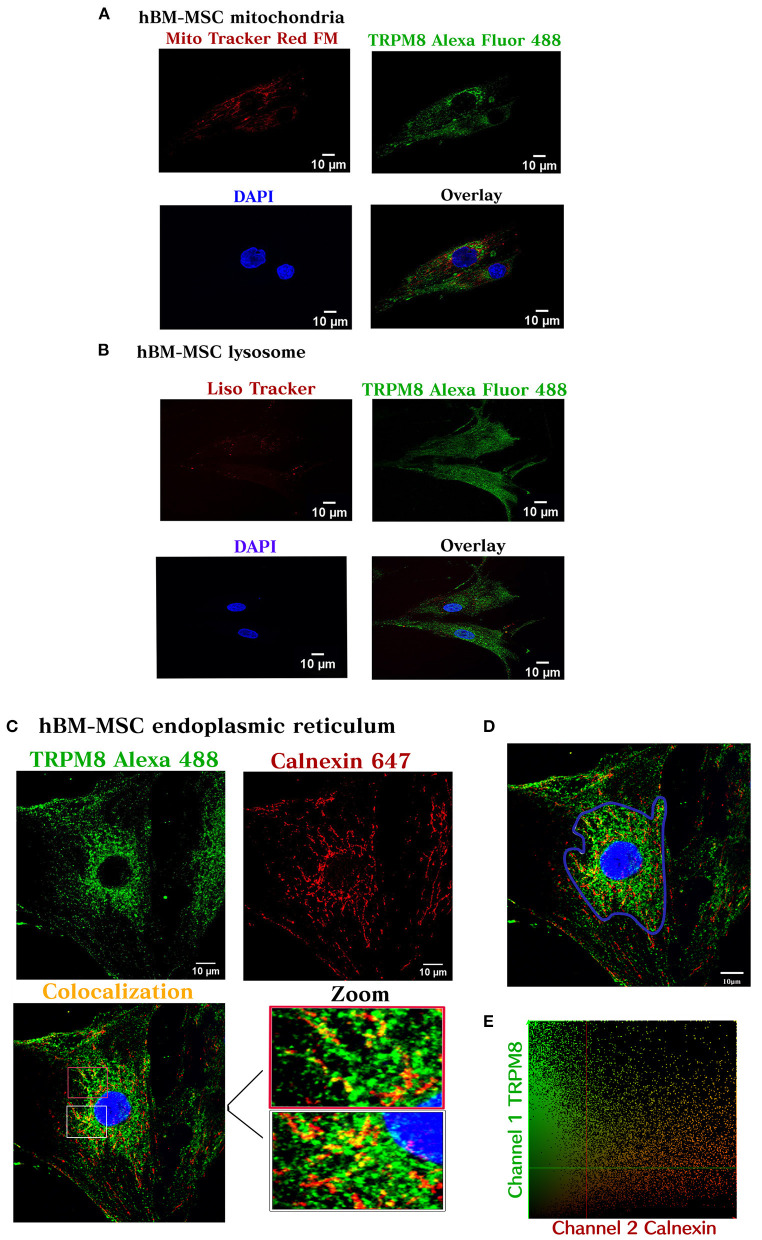
TRPM8 hBM-MSC localization. Maximum image volume projection of hBM-MSCs after double staining with the antibody TRPM8 conjugated with Alexa 488 and Mitotracker Red FM (colocalization index 0.03) (Overlap 0.36) **(A)**, Lisotracker Red (colocalization index 0.02) (overlap 0.49) **(B)**, and Alexa 647 calnexin (colocalization index 0.14) (Overlap 0.60) **(C)**. **(D)** Selected area for the colocalization analysis (perinuclear region). **(E)** Colocalization analysis (*n* = 3) experiments.

Next, we tested the subcellular pattern distribution of the channel in hBM-MSC, using immunofluorescent confocal microscopy in permeabilized cells. Results revealed that TRPM8 is endogenously expressed on hBM-MSC with a diffuse distribution throughout the cell (Figure 2D) (*n* = 4). As shown in Figure 2D, the TRPM8 signal is preferentially located at the plasma membrane (PM) and cytoplasm with perinuclear expression (see arrows). To better appreciate the channel localization, we performed double staining using the TRPM8 antibody, fluorescent probes to visualize mitochondria (Mitotracker) and lysosomes (Lisotracker), and calnexin antibody to visualize colocalization in the endoplasmic reticulum (ER). Using Pearson's correlation and overlap coefficient as colocalization parameters, we did not observe colocalization in mitochondria and a low colocalization in lysosomes (Figures 3A,B). However, TRPM8 colocalized with ER protein calnexin, suggesting that the TRPM8 channel can be localized in proximity to the ER (Figure 3C). This colocalization was mainly restricted to the perinuclear region supporting localization of the TRPM8 channel in ER (Figure 3D) (*n* = 3). The points of the scatterplot cluster reflect the ratio of the fluorescence of the two probes; also indicated the colocalization index and overlap of the perinuclear events (Figure 3E).

### hB-MSCs Express Functional TRPM8 Channels

**Figure 4:**
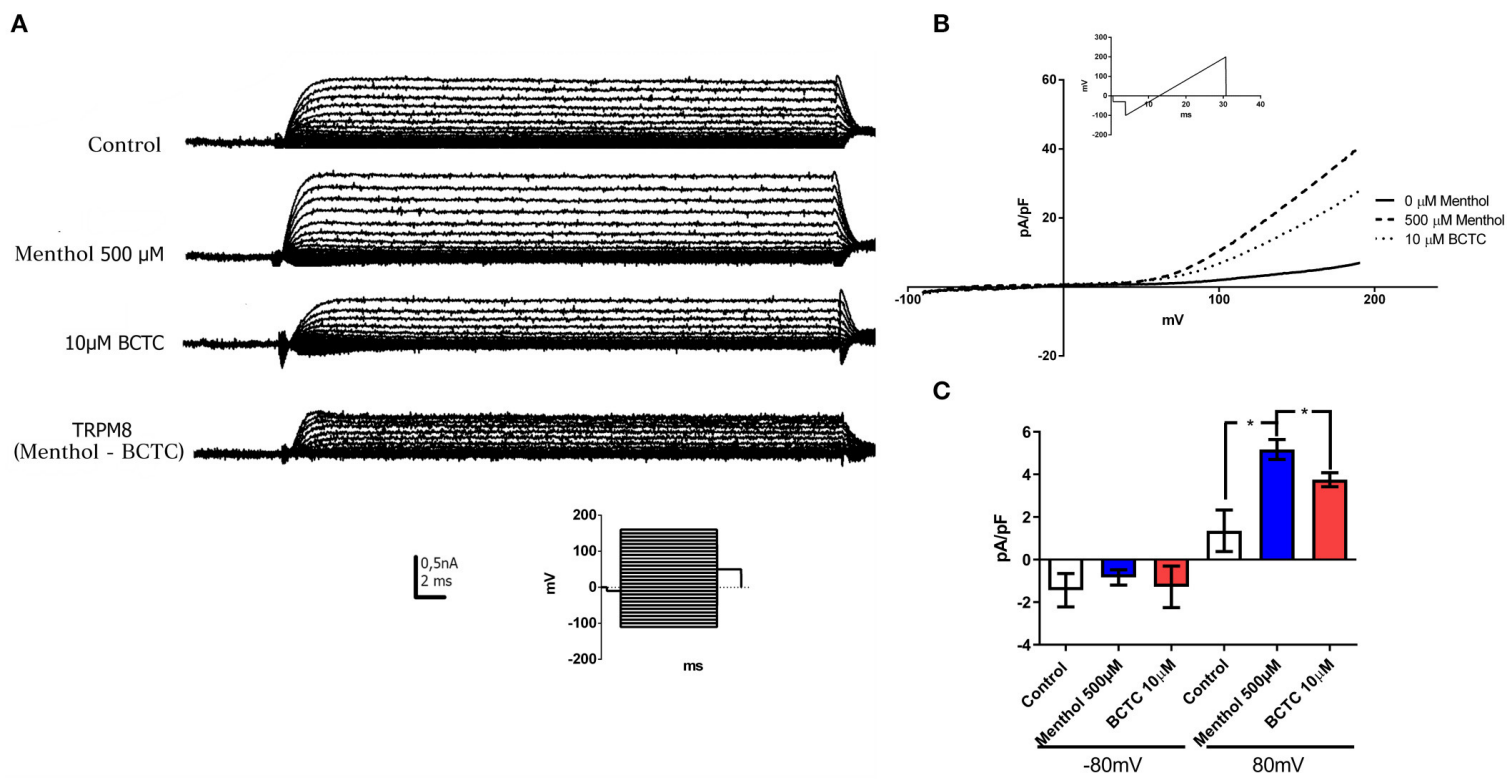
hBM-MSCs express functional TRPM8 channels. **(A,B)** Currents evoked in response to a voltage pulse protocol. Current- voltage (IV) curves in whole cell configuration in hBM-MSCs cells before (control) and after the addition of menthol 500 μM and BCTC 10 μM (*n*: 4). (Insert: Voltage step protocol). TRPM8 currents were calculated by subtracting the residual current after the addition of BCTC current from the current activated by menthol. **(B)** IV in response to a voltage ramp protocol in configuration of whole cell before (control, continues line) and after the addition of menthol 500 μM (dashed line) and BCTC 10 μM (dotted line) (*n* = 4). The ramp protocol used for the acquisition of the records is indicated in the insert. **(C)** Current density (pA/pF) from hBM-MSC at −80 mV and +80 mV, before and after menthol addition. *P < 0.05. Data are mean ± SD (*n* = 3).

TRPM8 protein forms non-selective cationic channels. Thus, patch-clamp recordings in whole-cell configuration were performed to measure currents in the presence of TRPM8 agonists and antagonists (Figures 4A–C). 500 μM menthol applied in the bath induced an increase in the current in response to a step voltage protocol, as reported for TRPM8 (McKemy et al., 2002). The current was partially abolished after the addition of TRPM8 antagonist BCTC (10 μM) (Figure 4A). The analysis of total current density by the response to a voltage ramp protocol, showed that the application of menthol produced a clear augmentation of the outward current at depolarized potentials (Mälkiä et al., 2007) (Figure 4B). The current density evaluated at +80 mV increased in ~3.8 times (control 1.35 ± 0.97pA/pF; Menthol 5.17 ± 0.81 pA/pF) (*n* = 3) (Figure 4C) after menthol addition. Twenty six percent of the menthol activated currents were abolished after the exposure to the TRPM8 antagonist BCTC (Figure 4C). We did not observe any effect on the inward currents (evaluated at −80 mV) after the addition of menthol or BCTC. Our results showed that hBM-MSC express menthol-activated and BCTC-sensitive currents with a strong rectification, similar to reported previously for TRPM8 channels in other native cells, suggesting that hBM-MSC express functional TRPM8 channels.

### Activation of TRPM8 Channel Activity Promotes hBM-MSC Differentiation to the Osteogenic Linage

**Figure 5:**
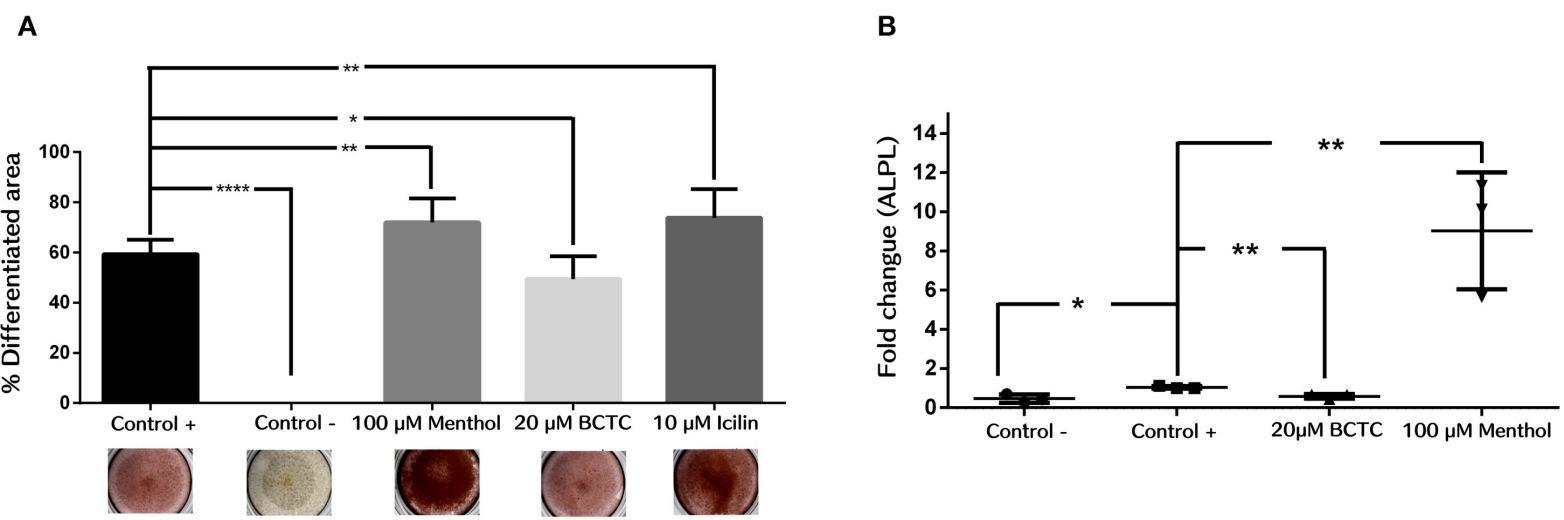
Osteogenic differentiation in hBM-MSCs cells is regulated by exposure to TRPM8 channel modulators. **(A)** Percentage of differentiated area from hBM-MSCs, detected with Alizarin Red (n = 3). Negative control (Control -), ****P < 0.0001; 100 μM menthol, **P < 0.001; 20 μM BCTC, *P < 0.01; 10 μM Icilin, **P < 0.001; (n = 3). **(B)** Rate of change measured by qPCR of the ALPL gene after 14 days of osteogenic differentiation; Negative control (Control -), *P < 0.01; BCTC 20 μM, **P < 0.001; 100 μM Menthol, **P < 0.001.

Next, we evaluated the effect of TRPM8 activity modulation in the differentiation to osteogenic lineage of hBM-MSC. Then, we induced osteogenic differentiation by adding osteogenic differentiation media in the presence and absence of the agonist menthol or icilin (100 and 10 μM, respectively), or the antagonist BCTC (20μM). We evaluated the efficiency of differentiation by staining calcium (Ca^2+^) deposits in differentiated cells, with the alizarin red dye. The results showed that the agonists menthol and icilin had a positive effect on osteogenic differentiation, as the production of Ca^2+^ deposits was increased in the presence of both agonists. Results showed that after treatment with menthol and icilin, the differentiated area increased in near 20% (control 59.34 ± 5.71; Menthol 72.02% ± 9.6; Icilin 73.86% ± 11) (*n* = 3) (Figure 5A). In contrast, after treatment with the antagonist BCTC, fewer deposits were observed (near to 10%) (Control 59.34% ± 5.71; BCTC 49.48% ± 9.0) (*n* = 3). These results suggest that activation of TRPM8 channels increase the osteogenic differentiation. On the contrary, negative modulation of the channel induced a decrease in osteogenic differentiation. To confirm those results, we evaluated by qPCR the effect of the TRPM8 agonist and antagonist in the expression of the gene codifying for the protein ALPL, a marker of early osteogenesis (Figure 5B). We found a noteworthy increase in ALPL gene expression, after osteogenic differentiation of hBM-MSC in the presence of menthol 100 μM (Control 1.04 ± 0.07; Menthol 9.0 ± 2.9) (*n* = 3) (Figure 5B). In contrast, osteogenic differentiation in the presence of the antagonist BCTC prompted a decrease in ALPL gene expression (Control 1.04 ± 0.07; BCTC 0.59 ± 0.12) (*n* = 3) (Figure 5B). Together, these findings suggest that modulation of TRPM8 channel activity can influence osteogenic differentiation in hBM-MSC.

## Discussion

In this study, we assess the expression of the non-selective cationic channel TRPM8 in hBM-MSCs. We also evaluate its role during osteogenic differentiation. We found that TRPM8 is expressed in both PM and ER of hBM-MSCs, suggesting a role in the cellular homeostasis controlling intracellular ion fluxes. Similar results were previously reported in HEK, LNCaPs and PC12 cells, where TRPM8 was expressed in plasma and intracellular membranes (Zhang and Barritt, 2004; Thebault et al., 2005; Kayama et al., 2017). The channel expression was observed in lysosomal membrane of the catecolamina-secreting PC12 cell line, where the Nerve Growth Factor (NGF) increase total and cell-surface protein expression through a mechanism that involves phosphatidylinositol 3-kinase and p38 MAP kinase activity (Kayama et al., 2017). Although Zhang and Barritt (2004) reported TRPM8 expression in PM and ER of the prostate cancer-derived epithelial cell line (LNCaP)s, Thebault et al. (2005) found that the expression was restricted to ER, where it participated in Ca^2+^ store depletion after cold or menthol stimulation. That effect was associated with the control of proliferation, cell survival and apoptosis through the regulation of intracellular [Ca^2+^] (Zhang and Barritt, 2004; Thebault et al., 2005). The presence of TRPM8 in ER has been reported to be regulated by factors such as TRP-associated factors (TCAFs), that increase the protein trafficking to the PM in prostate cells. It has been proposed that TCAF1 and TCAF2 promotes the stabilization of the channel expression and control the TRPM8 channel activity in process such as cell migration (Gkika et al., 2015). Androgen receptor was also reported to modulate both expression and activity of the TRPM8 channel in a human prostate cancer cell line (PC3). That effect depend on the differentiation and oncogenic status of cells. Considering that we found a high intracellular expression of the channel, it could be interesting to analyze the regulatory mechanism involved in channel expression and activity, aiming to understand the role of TRPM8 regulating intracellular and PM ion fluxes in hBM-MSCs.

Electrophysiological characterization of the TRPM8-like currents showed an increase in current density after menthol addition, which was partially blocked by exposure to BCTC. Even though the percentage of current blockage by BCTC is 26%, we certainly believe that this current correspond to TRPM8. It has been suggest that mesenchymal cells express other TRP channels as TRPV1 (Ren et al., 2016). It is also known that menthol blocks TRPV1 (Takaishi et al., 2015). Therefore, since in our experiments we activate the channel by menthol is unlikely that TRPV1 is contributing to the measured total currents. Our results suggests the functional expression of TRPM8 channels in hBM-MSCs. In agreement with a low PM channel expression (Figure 2C), we observe a lower current density, compared with other cell types (Bidaux et al., 2007). TRPM8 channel activity is regulated by intracellular proteins and molecules that can modify channel expression, activity or biophysical properties (Gkika et al., 2015).

Considering the function that mesenchymal stem cells have in processes such as proliferation, migration and cell differentiation, which imply the activation of diverse mechanism, it is factible that channel expression or activity can be remodeled in different ways depending of the specific cell process. hBM-MSCs recorded currents had the typical outwardly currents reported for native and heterologously TRPM8 (McKemy et al., 2002; Peier et al., 2002; Brauchi et al., 2004; Voets et al., 2004; Raddatz et al., 2014), however, we could not observe any inward current as was previously reported for TRPM8 channels. In PC-3 cells have been shown that TRPM8 current properties change depending of the expression of the androgen receptor which increase the current density and change activation properties of the channel (Bidaux et al., 2007). There are not previous evidence of the TRPM8 channel expression in hBM-MSCs, therefore, the modulation of the biophysical properties of the channel by intracellular particular factors from hB-MSCs is unknown. Considering the previous reports, it is probable that hBM-MSCs TRPM8 currents could be regulated by the expression of specific molecules that change depeding on the cell stage of differentiation. Further studies would be required to address the regulation of TRPM8 in mesenchymal cells.

TRPM8 channels are expressed in neuronal cells, where they participate in nociception and temperature signaling (Gees et al., 2010). Further, TRPM8 channels are widely expressed in non-neuronal tissues including the bladder (Stein et al., 2004) and prostate cells (Valero et al., 2012). TRPM8 channels are also expressed and upregulated in some tumors including breast, lung and pancreatic carcinoma as well as glioblastoma, retinoblastoma and uveal melanoma cells (Nilius, 2007; Chodon et al., 2010; Yee et al., 2010; Mergler et al., 2012, 2014; Journigan and Zaveri, 2013). Moreover, TRPM8 inhibition and silencing reduce proliferative capacity in pancreatic adenocarcinoma and prostate cancer cells (Yee et al., 2010; Valero et al., 2012).

To the best of our knowledge, this is the first report about the expression of TRPM8 channels in hBM-MSCs. It has been described the role of ion channels in processes such as MSCs migration and differentiation (Louhivuori et al., 2009; Leng et al., 2015; Bidaux et al., 2016; Echeverry et al., 2016; Hong et al., 2020; Wang et al., 2020), therefore, we sought to evaluate if TRPM8 channels participate in hBM-MSCs differentiaton. Thus, we explored the correlation between the modulation of TRPM8 channel activity and osteogenic differentiation. We found a positive effect in the differentiation process with an increase in both differentiated area and ALPL gene expression when TRPM8 channels were activated with the channel agonists. Additionally, the differentiation was diminished when channel activity was decreased in response to the antagonist BCTC, which suggest a relation between TRPM8 channel activity and osteogenic differentiation. The function of diverse Ca^2+^ permeable TRP channels has been demonstrated to be important in BM-MSCs osteogenic differentiation. H_2_S production as initial step in osteogenic differentiation was related with the activation of TRPV3, TRPV6, and TRPM4 in hBM-MSCs. Moreover, it has been proposed that the increase in the expression of the osteogenic markers osteocalcin and Runx2 with the concomitant rise in osteogenic differentiation is triggered by increases in the intracellular [Ca^2+^] through the opening of Ca^2+^ channels. Rises in intracellular Ca^2+^ regulate the activity of protein kinase C (PKC) and extracellular-signal-regulated kinase (Erk) expression, downregulating the Wnt/β-catenin signaling (Gaur et al., 2005; Liu et al., 2014). TRPM7 expression has been shown to be increased during osteogenic differentiation in hBM-MSCs. Channel activity induces the phospholipase C (PLC) activation that promotes, through phospholipid phosphatidylinositol 4, 5-biphosphate (PIP_2_) hydrolysis, the generation of the Inositol 3-Phosphate (IP3). The IP3 binds to the ER IP3 receptor promoting Ca^2+^ release to the cytoplasm, which activates CAMK II and stimulates SMAD1 translocation to the nucleus to activate osteogenic genes RUNX2, Osterix and OCN (Xiao et al., 2014; Hong et al., 2020). The Ca^2+^ permeable mechanically activated TRPV4 channel has been demonstrated to increase the nuclear expression of the transcription factor NFATc1, which generate a rise in the osteogenic markers Osterix and ALP and prompts osteogenic differentiation in BM-MSCs (Hu et al., 2017). In keratinocytes, the modulation of the activity of the isoform epidermal of TRPM8 (eTRPM8) has been associated with the regulation of cell differentiation (Bidaux et al., 2015, 2016). Bidaux et al. (2015, 2016) demonstrated that eTRPM8 mediated Ca^2+^ ER release and mitochondrial Ca^2+^ uptake which promotes an increase in mitochondrial [Ca^2+^] and a rise in ATP and ${\text{O}}_{2}^{-}$ production. ATP and ${\text{O}}_{2}^{-}$ balance regulate proliferation and differentiation of keratinocytes (Bidaux et al., 2015). Here, we observed that activation of the Ca^2+^ permeable channel TRPM8 by menthol induced an increase in the ALP marker expression, in agreement with the increase in the osteogenic differentiated area. We also found that TRPM8 channel activation after temperature decreasing (from 37 to 24°C), promotes an increase in intracellular Ca^2+^ concentration (data no shown), suggesting a role of Ca^2+^ in the observed effects in cell differentiation. Blocking the TRPM8 activity eliminated the increase in ALPL expression and decreased osteogenic differentiation. As it has been established, the increase in intracellular [Ca^2+^] from both Ca^2+^ influx or ER depletion, is a common mechanism that activate different transcription factors involved in osteogenic differentiation in MSCs from diverse origins (Eapen et al., 2013; Koori et al., 2014; Sun et al., 2014; Tong et al., 2017; Yanai et al., 2019). As we shown, hBM-MSCs TRPM8 channels are mainly expressed in intracellular compartments and colocalization assays showed the expression of the protein in the ER, suggesting that its principal function could be related with Ca^2+^ release from intracellular compartments. In that sense, could be suggested that TRPM8 activation could promote a Ca^2+^ depletion from ER, which in turn activate the expression of transcription factors as ALPL and induce osteogenic differentiation. Future studies are required to determine the endogenous mechanism(s) that activate TRPM8 channels and the specific pathways that could be regulated by TRPM8 channel activity in hBM-MSCs.

TRPM8 is a cold-sensing channel activated by diverse mechanism including voltage, cold and both natural and synthetic agonist such as menthol and icilin (McKemy et al., 2002; Peier et al., 2002; Voets et al., 2007). TRPM8 agonist shifts its voltage-activation curve to negative potentials and promote the channel opening at physiological membrane potentials. On the contrary, antagonist of the channel change the voltage dependence to more positive potentials (Mälkiä et al., 2007). Considering that hBM-MSCs are not exposed to important temperature fluctuations, TRPM8 activation is quite likely to be induced by endogenous molecules such as the membrane PIP_2_ which was demonstrated to regulate TRPM8 gating without a thermal or chemical stimuli (Rohács et al., 2005). The lysophospholipids (LPSs) lisophosphatidylinosithol (LPI) and lisophosphatidylcholine (LPC) have been also reported as direct agonists of TRPM8 (Noyer et al., 2018).

Altogether, our data showed that TRPM8 channels are expressed in hBM-MSCs. Moreover, TRPM8 blockade using BCTC decreased the osteogenic differentiation, suggesting a role for TRPM8 in this process. However, assays knocking down the expression of TRPM8 (e.g., siRNA) are needed to further confirm the function of TRPM8 channels in hBM-MSCs osteogenic differentiation. Likewise, the use of additional markers could add relevant information to understand differentiation in human mesenchymal cells. Finally, the study of the proteins and factors that regulate differentiation of these cells will have important applications in regenerative medicine. Further studies to elucidate the regulation mechanisms of TRPM8 expression are required to the better understanding of the specific physiology and homeostasis of hBM-MSCs.

## Data Availability Statement

The original contributions presented in the study are included in the article/supplementary material, further inquiries can be directed to the corresponding author/s.

## Author Contributions

JH, AG, and CM-C performed research and analyzed data. AB and VR-P designed the experiments, analyzed data, and wrote the paper. IC, PR, and RL analyzed data and wrote the paper. EL-G and RP-N wrote the paper, YT designed the experiments, directed research activities, analyzed data, and wrote the paper. All authors contributed to the article and approved the submitted version.

## Conflict of Interest

The authors declare that the research was conducted in the absence of any commercial or financial relationships that could be construed as a potential conflict of interest.
